# Governing healthcare records of past medical injustice beyond consent-based research ethics: a cross-sectional survey of public attitudes toward purpose, proximity, and stewardship in Japan

**DOI:** 10.1186/s12910-026-01571-8

**Published:** 2026-08-12

**Authors:** Saori Watanabe, Motoyuki Goto, Tomonori Akashi, Yuki Suzuki, Yoko Matsubara

**Affiliations:** 1 Department of Public Policy, Institute of Medical Science, The University of Tokyo, 4-6-1 Shirokane-dai, Minato-ku, Tokyo, 108-8639 Japan; 2 Graduate School of Core Ethics and Frontier Sciences, Ritsumeikan University, 56-1 Toji-in Kitamachi, Kita-ku, Kyoto, 603-8577 Japan; 3 Kyushu University Archives, Kyushu University, 6-10-1 Hakozaki, Higashi-ku, Fukuoka, 812-8581 Japan; 4 College of Social Sciences, Ritsumeikan University, 56-1 Toji-in Kitamachi, Kita-ku, Kyoto, 603-8577 Japan

**Keywords:** Bioethics, Empirical bioethics, Public attitudes, Past medical injustice, Eugenic policy, Reproductive justice, Records governance, Health data governance, Secondary use, Consent

## Abstract

**Background:**

How can sensitive health information be governed when consent cannot provide the basis for its legitimate use? This question is acute for healthcare records created in contexts of historical injustice — such as forced sterilization and the segregation of stigmatized groups. Such records are indispensable for accountability and redress yet capable of inflicting renewed harm, while the people whose lives they document may be deceased, unreachable, or never have had a meaningful opportunity to consent. In Japan, the Supreme Court’s July 2024 ruling that the former Eugenic Protection Law was unconstitutional has sharpened these questions; however, public views on the conditions of legitimate preservation and use remain largely undocumented.

**Methods:**

We conducted a cross-sectional online panel survey in Japan in November 2024 (*n* = 1,143), using quotas for sex, age group, and prefecture among adults aged 20–69. Respondents reported their attitudes toward preservation and preferred custodians; the acceptability of six purposes of research use under three personal-proximity conditions (no personal information; self or living family members; ancestors); and their perceptions of benefits and risks. Variation by proximity and purpose constituted the primary comparison; demographic comparisons were exploratory and were summarized using Cramér’s V.

**Results:**

Most respondents (86.8%) supported preservation, but support was conditional, with opposition centered on uncertainty about future purposes and privacy; acceptance of research use varied by purpose and proximity. Medical research was the most widely accepted purpose (77.4% in the no-personal-information condition); humanities and social science research, citizen-led inquiry, and journalistic investigations were less widely accepted. Acceptance was lowest for records concerning respondents themselves or their living family members. Originating medical or welfare institutions received the strongest support as custodians. Women expressed greater concern about psychological risks, particularly the possibility of recalling painful past experiences (Cramér’s V = 0.13).

**Conclusions:**

Sensitive historical healthcare records can be governed neither by preservation imperatives alone nor by consent-based research ethics; their legitimate governance requires purpose-sensitive justification, relational privacy safeguards, and accountable stewardship. Public attitudes do not settle these normative questions but map the social conditions under which governance is likely to command legitimacy, indicating where explanation and trust-building are needed. They form one component of ethical legitimacy, complementing rather than replacing the moral perspectives of survivors, affected families, and disability communities.

**Supplementary Information:**

The online version contains supplementary material available at https://doi.org/10.1186/s12910-026-01571-8.

## Background

Records of past medical practice connected to historical injustice — those linked to forced sterilization, segregation, and other forms of state-enforced eugenic policy — pose a distinctive ethical challenge for societies confronting these histories. Such records are at once indispensable for accountability, redress, and historical understanding, and capable of inflicting renewed harm on those whose information they contain and on those connected to them through family, community, or generation. This challenge is now unfolding with particular force in Japan. The Eugenic Protection Law, enacted in 1948 and repealed in 1996, built on the wartime National Eugenic Law of 1940 [1] and authorized the sterilization of individuals deemed to have hereditary or mental disorders; 16,475 non-consensual sterilizations were recorded, alongside 8,518 procedures nominally performed with consent, with people with intellectual disabilities, mental illness, hereditary conditions, and Hansen’s disease disproportionately targeted. Women and girls underwent more than two-thirds of these non-consensual operations (11,312 of 16,475) [2] — a disparity suggesting that this injustice was rooted in gender as well as in disability and heredity. In July 2024, the Supreme Court of Japan ruled the law unconstitutional, recognizing it as a grave violation of human rights and affirming the state’s responsibility for reparations [3]. The ruling has strengthened the case for preserving and disclosing the primary records that document these abuses as a matter of public responsibility [4].

Yet the same reckoning has exposed two intertwined problems. The first is the large-scale loss and fragmentation of the relevant records: many eugenic surgery registers and application forms held by local governments were destroyed once their mandated retention periods expired, and a 2023 National Diet investigation could confirm only 4,878 of the 16,475 recorded non-consensual sterilizations (29.6%) from surviving local records, of which 3,022 contained identifying information [2]. The second is that the records that survive are ethically difficult to govern. Records of this kind hold highly sensitive information — illness, disability, reproductive history, institutionalization, and family relationships — whose preservation and secondary use may advance historical clarification, public learning, and the prevention of future abuses, yet may equally produce privacy infringement, stigma, discrimination, and renewed distress for survivors, descendants, and associated communities. Such records were generated not only in medical settings but also across the welfare facilities and public bodies through which these policies operated, so the governance challenge spans medical and welfare records alike. Existing legal and administrative frameworks have proven inadequate in both respects [5, 6].

These features place such records in a distinctive position for bioethical analysis [7]. As artifacts of healthcare encounters, they fall within the scope of medical ethics; yet their governance arises where prospective consent is structurally unavailable, where the people most directly recorded are typically unreachable, and where the very frameworks under which the records were created may themselves be objects of normative critique. The frameworks of clinical and research ethics — organized around contemporaneous consent, ongoing relationships, and forward-looking benefit — were not designed for this situation. The governing question is therefore not whether consent was obtained, but how legitimacy, privacy, and stewardship can be reconstructed once the conditions those frameworks assume are no longer available.

Two bodies of scholarship speak to this question but are rarely brought into dialogue. Within bioethics, the legitimate governance of sensitive health information has been theorized through accountable stewardship [8] — building on an earlier shift in genomic research ethics away from autonomy as the ultimate arbiter and toward the principles of reciprocity, mutuality, solidarity, citizenry, and universality [9] — as well as through relational rather than purely individual conceptions of autonomy and privacy [10, 11], the notion of a social license for data use [12], participatory arrangements such as data trusts and, more recently, data solidarity [13, 14], and, increasingly, the governance of the health data of deceased persons [15, 16]. Within archival studies, scholarship on records of structural violence has developed survivor-centered, trauma-informed, and harm-reduction approaches to their preservation and use [17–21]. In Japan, emerging work has begun to address the preservation and access conditions of medical archives [22]. An early focus-group study examined Japanese public and physician attitudes toward the secondary use of archived medical information and samples without specific consent [23], yet little is known about how the public perceives the long-term preservation and research use of records connected to past injustice. More broadly, public attitudes toward the secondary use of contemporary health data have been examined extensively, including in systematic and narrative reviews [24, 25], as well as in surveys of patient attitudes toward the research use of clinical data [26]. Recent syntheses draw their primary studies predominantly from high-income settings and suggest a recurrent but strongly context-dependent pattern in which public willingness, though frequently high, is conditional on privacy protection, transparency, trust, the purpose of use, and the data recipient. A 2024 systematic review of 116 studies (228,501 participants) identified privacy, security, and access to and control over data as recurring concerns across data types. It also found that perceived benefits relating to improvements in healthcare were a commonly reported motivation for sharing [27]. A 2025 review spanning 34 countries included a meta-analysis of more than 117,000 participants from 52 studies and similarly found that willingness was strongly recipient-dependent — just over three-quarters overall, but markedly higher for sharing with research organizations (80%) than with for-profit organizations using data commercially (25%) [28]. Determinants of willingness to share genomic data — particularly health literacy, trust, and perceived benefit — have themselves been the subject of recent review, with higher health literacy associated with fewer privacy concerns, and with trust — particularly institutional trust — often compensating for limited understanding [29]. This evidence, however, concerns data generated in contemporary clinical and research settings; comparable evidence on attitudes toward historical healthcare records, especially those connected to past medical injustice, remains, to our knowledge, scarce in the peer-reviewed literature.

These challenges are not unique to Japan. Comparable records, and active efforts to govern them, exist in other societies. In Germany, some 30,000 surviving patient files of victims of the Nazi “euthanasia” (T4) program — long believed lost and rediscovered in 1990 in the archive of the former East German Ministry for State Security — are preserved in the Federal Archives, where they have been catalogued to support historical research and the remembrance of victims as individuals [30]. In Canada, the Indian Residential Schools records compiled by the Truth and Reconciliation Commission are held by the National Centre for Truth and Reconciliation, which was established as a permanent archive to preserve the Commission’s records and to give Survivors and their communities access to their own history [31]; more broadly, Indigenous data-sovereignty frameworks such as the CARE Principles have been proposed as normative standards for the governance of Indigenous records and data [32]. In the United States, California’s twentieth-century eugenic sterilization records were reconstructed by researchers under institutional review board oversight to estimate the number of surviving victims and support a call for state redress [33]. The resulting database was subsequently specified in the state’s 2021 compensation statute as a means of verifying claimants’ identities [34]. Across these settings, the governance of records created in contexts of injustice is an active concern.

Public attitudes must, moreover, be situated carefully. The perspectives of survivors, affected families, and disability communities carry distinctive moral weight grounded in lived experience of harm, and the disability rights tradition has long held that decisions affecting a community cannot be made legitimately without that community’s participation [35]. Public attitudes can neither replace these perspectives nor confer ethical justification where affected communities themselves contest the preservation or use of such records. What they can do is indicate the broader social conditions under which governance is likely to be regarded as trustworthy by those not directly recorded [24], and reveal where public reasoning converges with, or departs from, the ethical commitments articulated in bioethics. This relevance is reinforced by evidence that public acceptance of sensitive data use is typically conditional on trust, transparency, and safeguards [25, 36].

Taken together, these strands point to an area that remains comparatively unexamined. The empirical evidence on public attitudes reviewed above largely concerns health data generated in ordinary clinical or research settings, and has rarely engaged records created in contexts of historical injustice — records whose subjects are typically deceased or unreachable, and for which prospective consent is unavailable. For such records, the relevant bioethical and archival scholarship has so far been largely conceptual, or grounded in the perspectives of survivors and affected communities, with comparatively little systematic evidence on how the wider public reasons about them. The present study addresses this gap. It provides population-level empirical evidence on public attitudes toward the long-term preservation and research use of sensitive historical healthcare records connected to past medical injustice, and reads that evidence through the contemporary bioethical concepts introduced above — stewardship, relational privacy, social license, and the governance of deceased persons’ data. In doing so, it seeks to connect debates on health-data governance and empirical bioethics to the ethical afterlives of records created in contexts of injustice. Although grounded in Japan, the questions it raises resonate internationally.

Against this background, this study examines public attitudes relevant to the ethical governance of sensitive historical healthcare records in Japan through an online survey of the general public. The 2024 ruling provides a motivating context rather than the study’s exclusive object: the questions at issue extend to historical healthcare records whose preservation and secondary use are ethically important but socially sensitive. We ask three questions: whether the public supports the long-term preservation of such records; how acceptance of their use varies by intended purpose and by the personal or familial proximity of the information; and what benefits, risks, and custodial arrangements respondents associate with legitimate governance. We adopt a stratified approach to empirical bioethics, distinguishing three levels: descriptive findings, their interpretation through normative resources, and data-informed proposals that bring the two together [37]. On this view, empirical findings cannot settle normative questions, but they can map the social conditions under which governance arrangements may or may not command legitimacy. The survey responses thus bear on a broader question: how such records can be governed once neither preservation imperatives nor consent-based research ethics alone provides a sufficient basis.

## Methods

### Study design and definition

In this article, “historical healthcare records” refers to records created in healthcare contexts, including those held by medical institutions, welfare facilities, and related public bodies. This study employed a cross-sectional online survey to examine public attitudes toward the long-term preservation and research use of historical healthcare records in Japan. The survey was designed to explore views on the preservation of such records, acceptable purposes of research use, preferred custodial institutions and retention periods, and perceived risks and benefits associated with their use.

### Participants and sampling

The survey was conducted between 6 and 8 November 2024 using an online questionnaire. Participants were recruited from the panel of MIGHTY Monitor, managed by INTAGE Inc., a major Japanese survey research company. Invitations were distributed to registered panel members aged 20 to 69 years. Sampling quotas were set to approximate the national distributions of sex, age group, and prefecture of residence among adults aged 20–69. Individuals working in occupations closely related to media, advertising, think tanks, or healthcare and welfare services were excluded in order to reduce potential professional bias in responses.

A total of 10,950 individuals were invited to participate, and 1,143 valid responses were obtained, yielding a participation rate of 10.4%. The mean completion time for valid respondents was 9.4 min. Two structural features of this sampling design should be noted. First, online consumer panels systematically under-represent individuals without regular digital access, those in long-term institutional care, and members of communities historically subject to medical segregation — populations whose perspectives are particularly germane to the topic of historical healthcare records. Second, the panel composition was designed to approximate the general population by sex, age, and prefecture, but not by educational attainment, disability status, or any indicator of personal or familial connection to the histories under examination. Findings should therefore be read as exploratory indications of broad public reasoning rather than as representative of the views of those most directly affected.

### Survey instrument and measures

Before completing the questionnaire, respondents were provided with an explanatory text describing what historical healthcare records might include. The questionnaire then covered respondents’ familiarity with the concept of archives, views on whether historical healthcare records should be preserved, preferred retention periods, preferred custodial institutions, and perceptions of the benefits and risks of using such records for research. To examine how acceptance of research use varies with the personal proximity of the information, three conditions were presented sequentially in the order shown to respondents: (i) records containing no personal information (e.g., hospital bed-count statistics, occupancy records of welfare facilities, administrative records); (ii) records containing information about the respondents themselves or their living family members (e.g., physicians’ clinical notes, case records held by welfare facilities); and (iii) records containing information about the respondents’ ancestors (e.g., clinical records of great-grandparents, or medical records from roughly a century ago). These conditions differ along several correlated dimensions rather than along a single graded scale: whether the respondent is included, whether the persons concerned are living or deceased, and how temporally distant the records are. We therefore use “personal proximity” as a summary label for this set of conditions, not as a unidimensional measure. For each of these three conditions, respondents indicated whether each of six possible purposes of research use was acceptable: medical research; social welfare research; humanities and social science research (such as historiography and sociology); citizen-led inquiry; journalistic investigations; and educational use (e.g., student research and classroom learning). Respondents could select multiple purposes. The questionnaire also included a small number of general-attitude and background items presented at the outset — prior use of record-holding facilities, interest in health- and welfare-related topics, and general acceptability of selected contemporary technologies — that are not analyzed here. The full questionnaire as administered, including these items, together with summaries of the explanatory and framing materials shown to respondents, is provided in Additional file 1.

### Statistical analysis

Descriptive statistics were used to summarize respondents’ views on preservation and research use. The primary comparisons were defined by the study design: variation in acceptance across the three proximity conditions and across the six purpose categories. These comparisons were treated as pre-specified descriptive contrasts rather than inferential tests of independent groups, since the three proximity conditions were answered by the same respondents. Cross-tabulations were also used to explore differences by sex, age group, and educational attainment. Pearson’s chi-square tests were used to assess associations, and Cramér’s V was reported to indicate their magnitude. For multi-response items, respondents could select several options; associations with demographic variables were therefore assessed separately for each option as a respondent-level binary indicator (selected vs. not selected), rather than by a single joint test across options. To aid interpretation, selected proportions and differences in proportions are presented with 95% confidence intervals (CIs; Wilson intervals for proportions and Wald intervals for differences in proportions). Differences in acceptance across these within-subject conditions were also assessed with Cochran’s Q and pairwise McNemar tests with Holm adjustment. Because the demographic comparisons were exploratory, we emphasize effect sizes (Cramér’s V) alongside p-values and additionally report Benjamini–Hochberg false discovery rate (FDR)-adjusted results; associations are described as robust only where they remain significant after this adjustment. Cross-tabulations and association statistics, including Cramér’s V and non-significant comparisons, are reported in Additional file 2. All analyses were conducted using IBM SPSS Statistics version 30.0.

### Ethics and consent

The study used an anonymous online panel survey administered by INTAGE Inc.; the authors received only anonymized response data. It was conducted in compliance with applicable Japanese laws and research ethics guidelines, including the Code of Ethics of the Japan Sociological Society [38] and the Marketing Research Code of the Japan Marketing Research Association [39]. As an anonymous survey that did not involve biomedical intervention, biological samples, clinical records, or directly identifying personal information, it did not require ethics committee review under the Japanese government’s Ethical Guidelines for Medical and Biological Research Involving Human Subjects [40]. Before fielding, the questionnaire and the framing materials were reviewed by three external scholars with expertise in the history of Japan’s eugenic policies and forced sterilization records, to ensure that the subject matter was presented accurately and responsibly and that survey items were clearly and neutrally worded. Detailed ethics and consent procedures are reported in the Declarations section.

## Results

### Respondent characteristics

A total of 1,143 valid responses were included in the analysis (Table 1). The sample was almost evenly divided by sex, with 570 men (49.9%) and 573 women (50.1%). In terms of age, respondents in their fifties constituted the largest group (*n* = 282, 24.7%), followed by those in their forties (*n* = 250, 21.9%), sixties (*n* = 223, 19.5%), and those in their twenties and thirties (*n* = 194 each, 17.0%). With regard to educational attainment, university graduates formed the largest group (*n* = 395, 34.6%), followed closely by high school graduates (*n* = 390, 34.1%). Smaller proportions of respondents had completed vocational school (*n* = 121, 10.6%), junior/technical college (*n* = 112, 9.8%), or graduate school (*n* = 43, 3.8%), while 29 respondents (2.5%) reported middle school as their highest level of education. Fifty-three respondents (4.6%) selected “other” or declined to state their educational attainment. The age and sex composition of the sample broadly approximated that of the general Japanese population in the target age range [41]. Because educational attainment was not controlled for in sampling, analyses by education should be interpreted with corresponding caution.

**Table 1:** Characteristics of survey respondents (*n* = 1,143)

Characteristic	*n*	%
Sex		
Male	570	49.9
Female	573	50.1
Age group (years)		
20–29	194	17.0
30–39	194	17.0
40–49	250	21.9
50–59	282	24.7
60–69	223	19.5
Educational attainment		
Middle school	29	2.5
High school	390	34.1
Vocational school	121	10.6
Junior/technical college	112	9.8
University	395	34.6
Graduate school	43	3.8
Other/prefer not to answer	53	4.6
Total	**1**,**143**	**100.0**

Note. Age and sex composition broadly approximates the general Japanese population aged 20–69 [41]. Educational attainment was not controlled for in sampling. Percentages may not sum to exactly 100 due to rounding

The remaining results are organized around the three dimensions most relevant to the ethical governance question: support for preservation, acceptance of use across purpose and personal proximity, and respondents’ views about custodianship, benefits, and risks.

### General support for the preservation of healthcare records

**Fig. 1:**
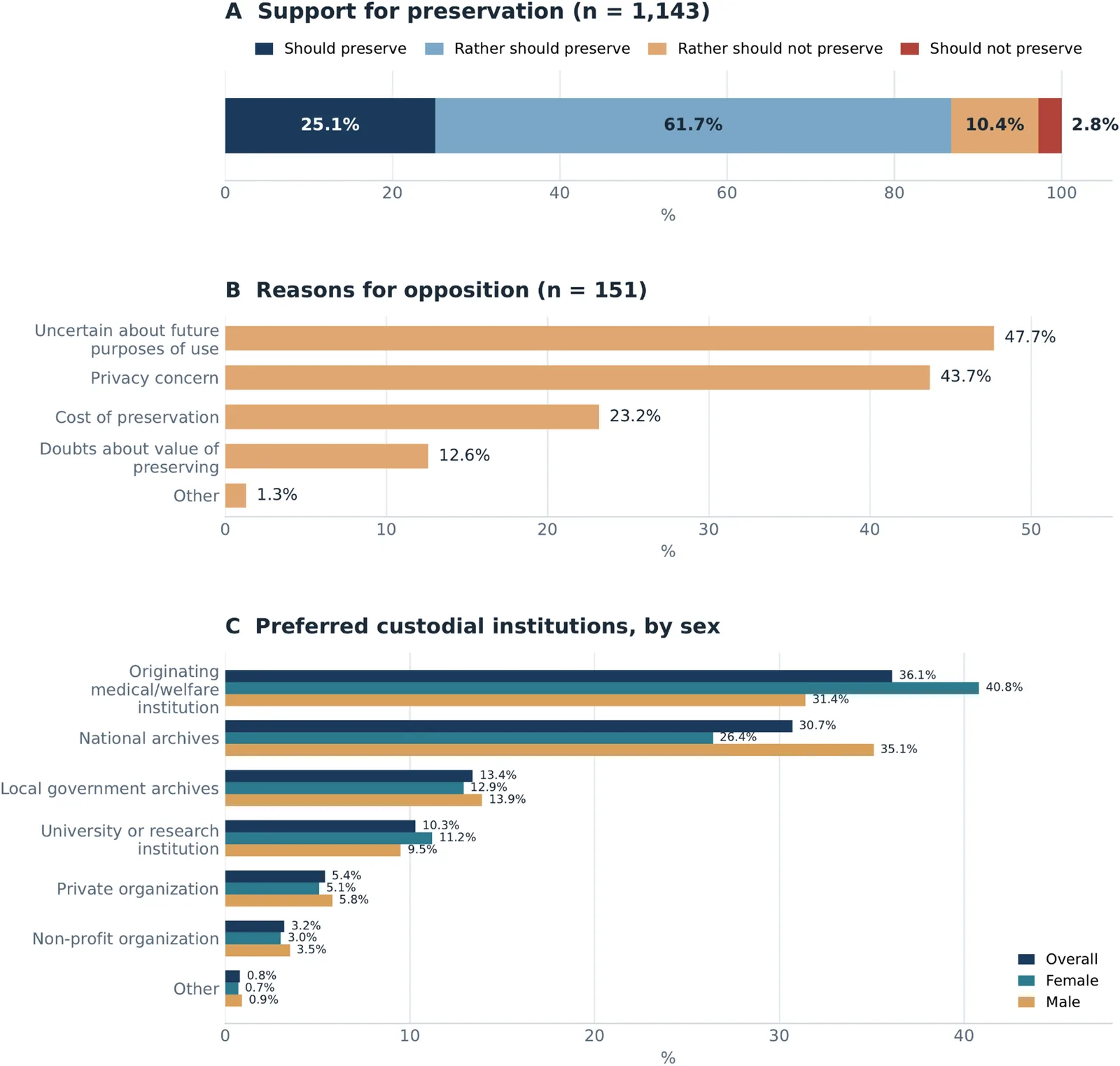
Public support for preservation, reasons for opposition, and preferred custodial institutions. Note. Panel **A**: support for preservation (*n* = 1,143); support = “should preserve” + “rather should preserve” (86.8%), opposition = “rather should not” + “should not” (13.2%); differences by sex were small (Cramér’s V = 0.08). Panel **B**: reasons for opposition, based on respondents who opposed preservation (*n* = 151). Panel **C**: preferred custodial institutions by sex; distributions differed by sex before FDR correction (Cramér’s V = 0.12; this difference did not survive FDR correction), with women more likely than men to prefer the originating medical or welfare institution and men more likely to prefer the national archives; differences for the remaining categories were small. Percentages may not sum to 100 due to rounding

Overall, 86.8% of respondents supported the preservation of historical healthcare records (25.1% “should preserve”; 61.7% “rather should preserve”), whereas 13.2% expressed some degree of opposition (10.4% “rather should not preserve”; 2.8% “should not preserve”), with no substantive difference by sex (Cramér’s V = 0.08; see Additional file 2 for full statistics). Among those who opposed preservation, the most frequently selected reason was uncertainty about the purposes for which the records might later be used (47.7%), followed closely by concerns about privacy protection (43.7%). By contrast, fewer respondents cited the cost of preservation (23.2%) or doubts about the value of preserving such records (12.6%) as reasons for opposition. These findings suggest that opposition to preservation more often reflected concerns about future use and privacy protection than doubts about preservation itself (Fig. 1A, B).

### Preferred retention periods and custodial institutions

Regarding retention periods, the most frequently selected option was 10 years (36.0%), followed by 50 years (20.7%), while 18.4% supported permanent preservation. Preferred retention periods thus varied widely.

With respect to custodial institutions, the most frequently selected option was the originating medical or welfare institution (36.1%), followed by the national archives (30.7%) and local government archives (13.4%). Universities and research institutions were selected by 10.3% of respondents, whereas non-profit organizations and private organizations attracted comparatively little support (3.2% and 5.4%, respectively). The distribution of preferred custodians differed by sex (Cramér’s V = 0.12): women were more likely than men to prefer the originating medical or welfare institution (40.8% vs. 31.4%; difference 9.4% points, 95% CI 3.9–15.0), whereas men were more likely than women to prefer the national archives (35.1% vs. 26.4%; difference 8.7% points, 95% CI 3.4–14.1); this overall sex difference in custodial preference, however, did not survive FDR correction and should be interpreted with caution. Differences for the remaining categories (local government archives, universities and research institutions, non-profit organizations, private organizations, and other) were small. Prior familiarity with the term “archives” was also limited: 18.1% of respondents reported knowing the term, a further 36.5% had heard it without understanding it, and 45.4% had no prior knowledge. Knowledge of the term was associated with educational attainment (Cramér’s V = 0.22; Additional file 2). These findings suggest that respondents distinguished not only whether records should be preserved but also which institutions were regarded as appropriate custodians (Fig. 1C).

### Acceptance of research use under different conditions

**Fig. 2:**
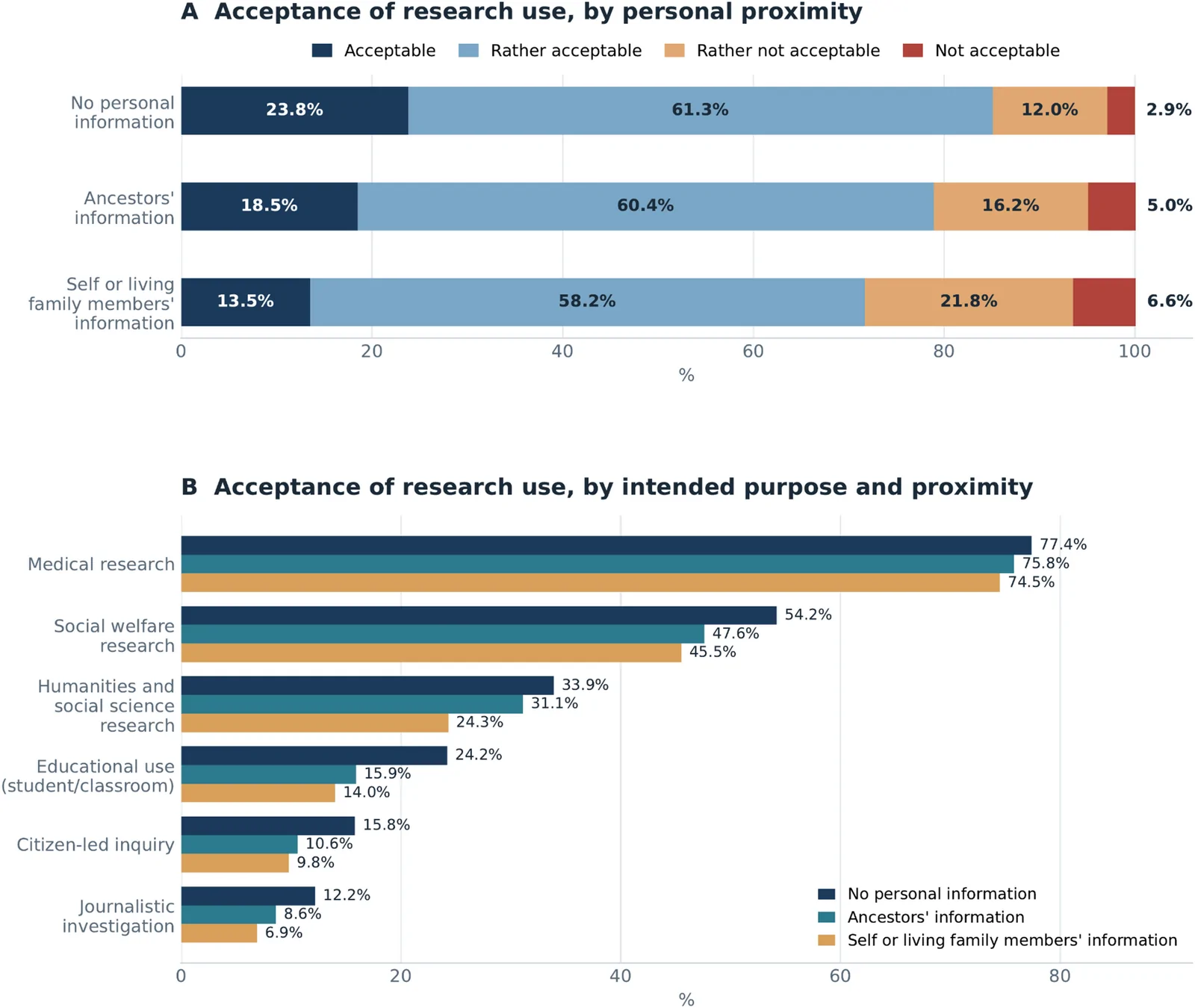
Acceptance of research use of historical healthcare records, by personal proximity and intended purpose. Note. *n* = 1,143. Panel **A**: acceptance of research use by the personal proximity of the information (no personal information; ancestors’ information; self or living family members’ information), shown in descending order of acceptance rather than the order of administration described in the Methods; a tentative sex difference before FDR correction was observed when records implicated respondents themselves or their living family members (Cramér’s V = 0.10), and differences by educational attainment across the three proximity conditions are reported in Additional file 2. Panel **B**: acceptance by intended purpose under each proximity condition; multiple responses permitted. “None of the above” responses: no personal information 12.9%; ancestors’ information 15.7%; self or living family members’ information 17.4%. Percentages may not sum to 100 due to rounding

Acceptance of research use varied according to the perceived personal proximity of the records. When records were described as containing no personal information, 85.1% of respondents (95% CI 82.9–87.1) indicated that such use would be acceptable, whereas 14.9% expressed some degree of opposition. Acceptance declined when the records were described as including information about the respondents themselves or their living family members: in this condition, 71.7% (95% CI 69.0–74.2) expressed acceptance and 28.3% opposition. Records including information about ancestors were viewed somewhat more favorably than records involving oneself or living family members, with 78.8% (95% CI 76.4–81.1) indicating acceptance and 21.2% opposition. Differences across the three conditions were statistically significant (Cochran’s Q = 129.5, df = 2, *p* < 0.001), with each pairwise contrast remaining significant after Holm adjustment. These results suggest that acceptance of research use was shaped not only by the general concept of using such records, but also by the perceived personal and familial proximity of the records (Fig. 2A).

Differences by sex were also observed. When records were described as including information about respondents themselves or their living family members, women were less accepting than men (Cramér’s V = 0.10): women were less likely than men to select “acceptable” (10.6% vs. 16.3%; difference 5.7% points, 95% CI 1.7–9.6) and more likely than men to select “rather not acceptable” (25.0% vs. 18.6%; difference 6.4% points, 95% CI 1.6–11.1). A similar but smaller pattern was observed for records including information about ancestors (Cramér’s V = 0.07); neither sex difference in acceptance remained significant after FDR correction, and both should be treated as tentative. Acceptance of research use in the no-personal-information condition did not differ appreciably between men and women (Cramér’s V = 0.04). Differences by educational attainment were evident across all three proximity conditions, with respondents holding a university degree or higher tending to express greater acceptance. In a comparison of respondents with a university degree or higher and those with less education (excluding the small numbers who selected “other” or declined to state; *n* = 1,090), Cramér’s V was 0.17 in the no-personal-information condition, 0.10 in the self-or-living-family condition, and 0.12 in the ancestors condition; full cross-tabulations across all educational categories, together with these dichotomized comparisons, are reported in Additional file 2.

### Differences by intended purpose of use

Respondents clearly differentiated among possible purposes of use across all three conditions. When records were described as containing no personal information, medical research was accepted by 77.4% of respondents, followed by social welfare research (54.2%), humanities and social science research (33.9%), educational use (24.2%), citizen-led inquiry (15.8%), and journalistic investigations (12.2%). When records implicated respondents themselves or their living family members, acceptance was consistently lower: medical research was accepted by 74.5%, social welfare research by 45.5%, humanities and social science research by 24.3%, educational use by 14.0%, citizen-led inquiry by 9.8%, and journalistic investigations by 6.9%. For records concerning ancestors, acceptance generally fell between these two conditions; for most purposes it lay closer to the self-or-living-family condition, while remaining slightly higher than in that condition across all purposes. The proportion indicating that none of the listed purposes would be acceptable also varied across conditions: 12.9% in the no-personal-information condition, 15.7% in the ancestors condition, and 17.4% in the self-or-living-family condition. These findings suggest that respondents evaluated not only whether such records should be used, but also the legitimacy and social purpose of each intended use. This evaluation was further shaped by the personal proximity of the records (Fig. 2B).

### Perceived benefits and risks

Respondents recognized both public benefits and ethical risks in the research use of historical healthcare records. Among the perceived benefits, the most frequently selected item was the possibility that society as a whole could share lessons from the past through research based on such records (55.7%), followed by the possibility of revealing historical realities that medical or welfare institutions might not wish to disclose on their own (40.7%). Other frequently selected benefits included contributing to safer and fairer contemporary policies for patients and families (36.6%), the possibility that victims of medical human rights violations could draw on a wider range of materials in legal proceedings (31.9%), a more accurate understanding of the experiences of patients and their descendants during the period in question (30.7%), and improving understanding of discrimination and prejudice experienced by patients and their families (28.1%). A further 20.1% selected the possibility that citizens and patients would have greater opportunities to learn about their own experiences. In addition, 19.5% of respondents indicated that none of the listed benefits applied.

By contrast, the most frequently perceived risk was concern about privacy protection and the leakage of personal information (52.5%). This was followed by concern that states, companies, or medical institutions might conceal or destroy information in order to avoid disadvantage or responsibility (36.6%), and concern that past events might become clearer in ways that could expose patients’ families or descendants to discrimination or prejudice (29.6%). A further 23.1% selected the possibility that some people might not wish to recall painful past experiences or might prefer the past to be forgotten. Another 21.2% considered that the costs of maintaining archival facilities would not be justified by the research outcomes obtained, and 16.5% expressed concern that physicians or medical institutions might be held responsible for past events. In addition, 8.4% indicated that past medical and welfare events were not personally relevant to them, and 19.9% indicated that none of the listed risks applied.

**Fig. 3:**
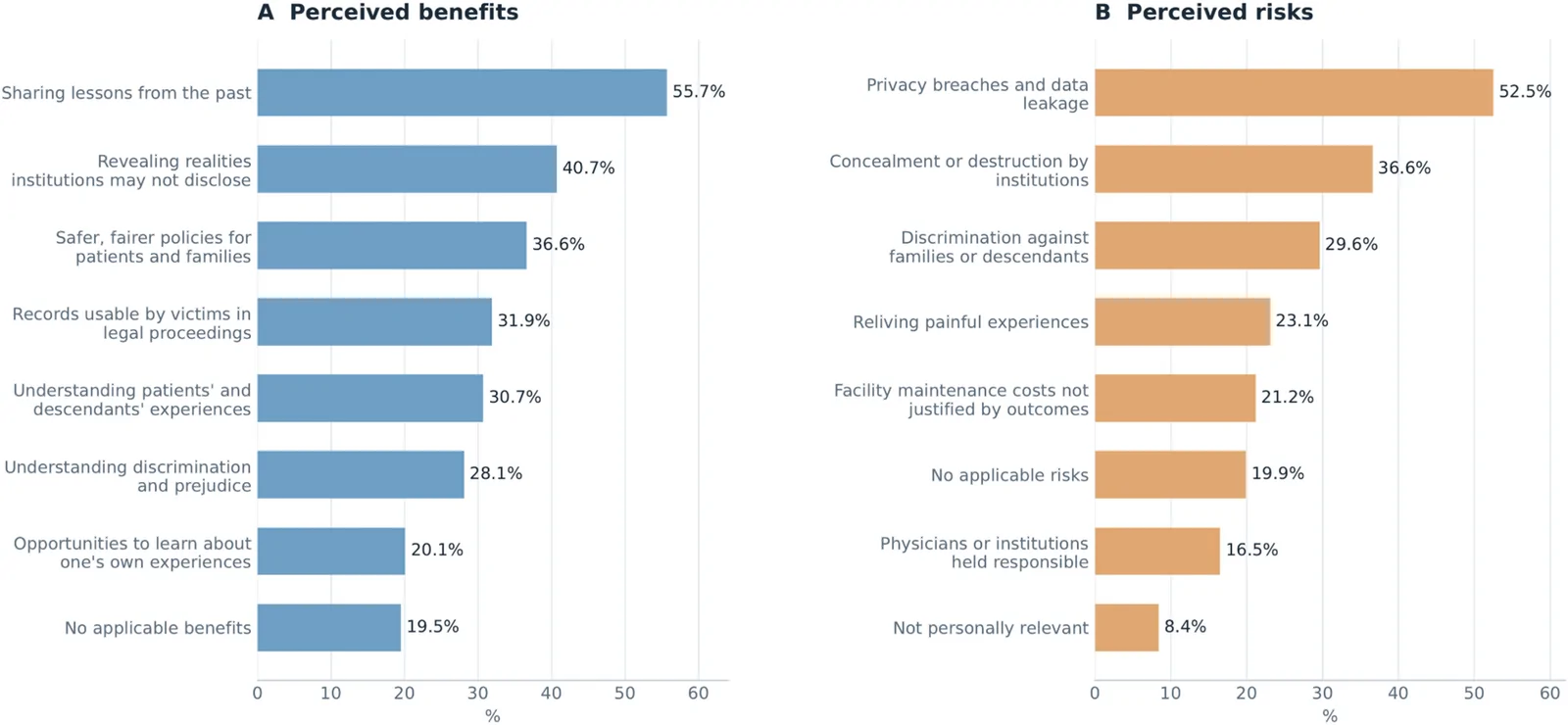
Perceived benefits and risks of research use of historical healthcare records. Note. *n* = 1,143; multiple responses permitted. Panel **A**: perceived benefits. Panel **B**: perceived risks. Differences by sex were more pronounced for risks than for benefits; the largest effect concerned the possibility that painful past experiences might be recalled (Cramér’s V = 0.13; see Additional file 2). Percentages exceed 100 because multiple responses were permitted

Differences by sex were more pronounced for perceived risks than for perceived benefits. Responses to most benefit items were broadly similar between men and women; for example, the item concerning safer and fairer contemporary policies for patients and families was selected by the same number of men and women (*n* = 209 each; 36.7% of men and 36.5% of women). One tentative exception before FDR correction was the possibility that victims of medical human rights violations could draw on a wider range of materials in legal proceedings, which women were modestly more likely than men to endorse (Cramér’s V = 0.08). Of the four risk items showing statistically significant sex differences before FDR correction, three were selected more often by women. Women more frequently selected the item concerning the possibility that painful past experiences might be recalled — the largest effect among the risk items (28.4% vs. 17.7%; difference 10.7% points, 95% CI 5.9–15.6; Cramér’s V = 0.13). Smaller differences in the same direction were observed for the item concerning privacy and data leakage (55.7% vs. 49.3%; difference 6.4% points, 95% CI 0.6–12.2; Cramér’s V = 0.06) and for the view that the costs of maintaining archival facilities are not justified by the research outcomes (24.1% vs. 18.2%; difference 5.8% points, 95% CI 1.1–10.6; Cramér’s V = 0.07). Of these three differences, only women’s greater concern about recalling painful experiences remained significant after FDR correction. In addition, one difference in the opposite direction survived correction: men were more likely than women to regard past medical and welfare events as not personally relevant to them (men 11.6% vs. women 5.2%; Cramér’s V = 0.11). Concern that information might be concealed or destroyed to avoid disadvantage was also selected more frequently by women, though the difference was smaller and not statistically significant (women 39.1% vs. men 34.0%; difference 5.1% points, 95% CI −0.5 to 10.6). This overall pattern suggests that women tended to perceive psychological risks, particularly the recall of painful experiences, more strongly than men (Fig. 3).

## Discussion

Four findings from this survey bear on the ethical governance of sensitive historical healthcare records. First, public support for preservation was high but conditional, suggesting that preservation does not become legitimate simply because records are historically valuable. Second, acceptance of research use varied systematically by intended purpose, with medical research the most widely accepted and non-medical forms of inquiry — historical, sociological, journalistic, and citizen-led — less widely accepted. Third, acceptance also varied by the personal or familial proximity of the information, indicating that public acceptance is relational as well as purpose-sensitive. Fourth, respondents preferred originating medical or welfare institutions as custodians, which raises a stewardship problem. We treat these findings as one input to ethical reasoning, alongside the distinctive moral perspectives of survivors, affected families, and disability communities: they do not by themselves entail governance prescriptions, but they map where governance is likely to require explanation, justification, and trust-building if it is to be regarded as legitimate [37].

### Conditional legitimacy and the limits of consent

The findings show that members of the public in Japan were not opposed in principle to the preservation and research use of historical healthcare records, but that their support was conditional rather than unconditional. Opposition to preservation was driven chiefly by uncertainty about future purposes of use and concerns about privacy protection, rather than by doubts about the value of preservation itself. Preservation is therefore not perceived as ethically self-justifying; its legitimacy depends on how records are expected to be governed, accessed, explained, and used — a pattern consistent with broader research showing that public acceptance of the secondary use of health-related data is often conditional on trust, transparency, public benefit, and appropriate safeguards [25, 42].

This matters because records of past medical injustice often cannot be governed through the standard prospective consent model. Those most directly recorded may be deceased, unreachable, or never have had a meaningful opportunity to consent to the practices that produced the records. Legitimacy therefore has to be reconstructed through other means — purpose-sensitive access rules, accountable institutional roles, privacy safeguards, and dialogue with affected communities — and public attitudes, while they cannot settle these questions, indicate where governance is likely to require explanation, justification, and trust-building, extending debates on health-data governance to records whose subjects can no longer consent.

### Purpose-sensitive legitimacy

Respondents distinguished sharply among intended purposes: medical research was consistently the most widely accepted, whereas humanities and social science research, citizen-led inquiry, educational use, and journalistic investigations were accepted less often, across all three proximity conditions. This is ethically important because records of past medical injustice may be needed not only for biomedical research but also for historical inquiry, social analysis, journalism, legal accountability, and citizen-led efforts to recover suppressed histories; lower public acceptance of these uses does not make them illegitimate. Some accounts hold that individuals may have ethical reasons to contribute data to socially valuable research [43]. Such arguments do not, however, remove the need for a publicly defensible purpose and proportionate safeguards: within the social-value tradition in bioethics, social value is treated as an ethical requirement of research rather than as a matter settled by popularity [44]. Empirical studies of secondary-use attitudes point the same way: patient representatives have identified the absence of a specified purpose as the central deficiency of broad consent, treating purpose specification as a condition of acceptable use rather than an optional refinement [45]. The implication is that preservation and use should be tied to clear public purposes — accountability, research, education, and redress — whose relative weighting remains open to deliberation rather than fixed by administrative default. The strong preference for biomedical purposes therefore marks not a ranking of legitimacy, but precisely where governance must explain and justify less widely accepted forms of inquiry. Governance that simply tracked public acceptance would risk privileging biomedical research over forms of inquiry that are often central to accountability for past injustice.

### Relational privacy and personal proximity

Acceptance also varied with the perceived personal proximity of the records: it was highest when records contained no personal information, lowest when they concerned respondents themselves or their living family members, and intermediate when they concerned ancestors, a pattern that held across each category of intended purpose.

These conditions, however, do not isolate a single dimension of closeness. The self-or-living-family condition and the ancestors condition differ not only in proximity but also in whether the persons concerned are living or deceased, in temporal distance, and in how readily individuals could be re-identified. The conditions were also presented in a fixed order. The somewhat greater acceptance of research use for ancestral than for self-or-living-family records is therefore open to several readings that the present data cannot distinguish: a protective response toward oneself and identifiable living relatives, a relative discounting of the interests of the deceased [15], or an effect of question order. We therefore treat “personal proximity” as a convenient summary for this set of conditions rather than a measured unidimensional construct. Read cautiously, the pattern is perhaps best understood not as a simple gradient of distance but as sensitivity to the configuration of living and identifiable persons who may be exposed to harm.

What is at stake, then, is not only individual privacy but also relational harms, descendant interests, and intergenerational sensitivities — concerns that records linked to the former Eugenic Protection Law make acute, since such records may be indispensable for accountability and redress yet expose descendants and families to renewed vulnerability. Read in conversation with bioethics, this points beyond individualist accounts of privacy: work on relational autonomy emphasizes that interests and vulnerabilities are shaped by relationships rather than confined to isolated individuals [10, 11], and survivor-centered, trauma-informed, and harm-reduction approaches to archives converge on the same point [17–19]. An information-ethics account adds that the moral standing of health information need not lapse at death: protection is better keyed to context — sensitivity, identifiability, and the risk of harm to survivors — than to the category of data [46]. Access should therefore be calibrated to this relational configuration, with stronger safeguards where records implicate living, identifiable, or relationally connected persons. On a relational account, such calibration is warranted independently of public opinion; the specific thresholds, however, will require dialogue with those whose information is implicated.

### Accountability, harm, and gendered risk

The findings on perceived benefits and risks reinforce the same tension. Respondents recognized substantial collective benefits — especially sharing lessons from the past and clarifying realities that institutions might not otherwise disclose — and substantial risks, including privacy breaches, stigma affecting families or descendants, psychological burden, and institutional concealment. That a notable minority identified the potential for victims of medical human rights violations to use such records in legal proceedings points to a documentary value extending beyond historical understanding to redress, evidence, and accountability; yet disclosure can also expose individuals, families, professionals, and institutions to consequences that shape perceptions of legitimate governance.

Sex differences in reported perceptions were more pronounced for risks than for benefits, with women expressing greater concern about psychological harms in particular. Among the differences reflecting women’s greater concern, the largest — and the only one to survive correction for multiple comparisons — concerned the possibility of recalling painful past experiences (Cramér’s V = 0.13); the smaller difference in privacy-related concern did not survive correction. One plausible, non-specific interpretation of this pattern is the previously documented tendency for women to report higher perceived risk than men across many health, technological, and environmental domains [47]; on this reading, the present finding need not be specific to past medical injustice. At the same time, the injustices that motivate this study are themselves gendered: forced sterilization under the former Eugenic Protection Law disproportionately affected women [2], a pattern documented across twentieth-century eugenic programs, particularly in North America [48, 49], and the medicalized control of disabled women’s reproduction has long been central to feminist disability scholarship [50] and to debates on reproductive justice [51]. Whether the risk perceptions observed here are connected to these gendered histories, however, lies beyond what a general-population survey can establish; our data cannot distinguish a context-specific effect from the general gender gap in risk perception, and the reproductive-justice reading is therefore best treated as a theoretical implication to be examined through participatory and qualitative research rather than as a conclusion supported by these data. What the data do support is more modest but directly relevant to governance: public risk perception is not uniform, and governance arrangements should not assume a single shared threshold of concern.

The coexistence of accountability-related benefits and harm-related risks does not by itself determine how they should be balanced, but it indicates that bioethical analysis of these records cannot reduce the question to either accountability or non-maleficence alone: such records generate simultaneous, and sometimes competing, obligations to preserve evidence of injustice and to prevent renewed informational and relational harm.

### Stewardship beyond custodianship

Respondents did not regard all custodians as equally acceptable: the originating medical or welfare institution received the strongest support, followed by national and local government archives, while universities, non-profit, and private organizations attracted less support. This distinguishes support for preservation from trust in particular custodians, and it exposes a stewardship problem — in contexts of historical injustice, the institutions that created or held records may be socially familiar yet implicated in the practices under scrutiny, while bodies better equipped for independent preservation, access review, and historical accountability may lack public visibility or trust. As the Results show, awareness of preservation institutions distinct from originating facilities was low and stratified by education. The preference for originating institutions may therefore reflect not only familiarity but the comparative invisibility of independent preservation arrangements within the Japanese public’s institutional imagination. If so, the alternatives best suited to accountable preservation may be functionally absent from the choices respondents could meaningfully consider. Normatively, accounts of regulatory stewardship and of more collective models such as data trusts and data solidarity hold that custody of such records entails transparency, independent oversight, long-term preservation capacity, and mechanisms for affected communities to contest or shape access, irrespective of institutional familiarity [8, 13, 14]. Work on contemporary biobanks frames custodianship as an ongoing responsibility for governing access rather than as simple ownership [52]. We draw on this only by analogy: that work concerns living donors who have given consent, whereas the records considered here typically concern deceased persons who never could. This difference, if anything, sharpens the point — where consent can play no role, the legitimacy of custody rests on accountable stewardship alone. Our data cannot identify which institutions are best placed to meet these conditions; rather, they identify where trust is likely to be at issue and suggest that unfamiliar custodial models would require explicit explanation and trust-building — together with public-facing communication and civic education about preservation institutions, without which even well-designed independent arrangements may struggle to command public trust.

### Affected communities and the limits of public attitudes

Finally, governance should proceed through dialogue with affected communities. The disability rights tradition, survivor-centered archival scholarship, and frameworks of community data sovereignty hold that decisions about a community’s records cannot legitimately be made without that community’s participation — a normative condition that general public attitudes can neither satisfy nor override [17–19, 31, 32, 35]. Here, our findings mark the limit of their own reach: a general-public survey such as ours can map the broad social conditions of legitimacy but cannot substitute for the distinctive moral perspectives of survivors, affected families, and disability communities. The German, Canadian, and U.S. experiences noted earlier are consistent with this. In each, the governance of records created in contexts of injustice has developed beyond consent alone; and the Canadian case in particular, with its emphasis on Survivor-centered stewardship, illustrates how claims of community authority and self-governance can raise questions that public attitudes alone cannot settle. Governing the records of past medical injustice well, then, is less a matter of settling consent than of sustaining participation: it depends on the active involvement of affected communities, continuing dialogue among those with a stake in these records, and ongoing effort to build the broader public understanding and trust on which legitimate stewardship rests.

### Implications for governance

These findings do not, in themselves, prescribe any particular arrangement, and they cannot substitute for the participation of affected communities discussed above; but they do indicate several points at which the governance of sensitive historical records will need to be designed with care. Because support for preservation was conditional rather than unconditional, governance frameworks should not treat historical value as self-justifying: preservation needs to be tied to explicit, publicly defensible purposes, with transparency about how records will be accessed, used, and safeguarded [12, 44], consistent with evidence that public acceptance of secondary use is contingent on trust, transparency, and safeguards [25, 27, 28]. Because acceptance varied by intended purpose, access models should, we suggest, be purpose-sensitive — calibrating review and safeguards to the use at issue, in a manner consistent with broader data-solidarity approaches [14] — without thereby marginalizing forms of inquiry important to accountability and redress (historical, social-scientific, journalistic, and citizen-led) [17, 21], whose legitimacy rests on the public value of their purpose rather than on the level of endorsement they attract [43, 44]. Because acceptance varied with personal and familial proximity, access controls should be calibrated to the mix of living, identifiable, and closely connected parties that a given record implicates, with the strongest safeguards where that exposure is greatest [10, 11, 46]. Because respondents preferred originating institutions yet were largely unaware of independent preservation arrangements, institutional stewardship models should combine accountable, arm’s-length oversight and standing access review with the public communication and civic education discussed above [8, 13]; where the originating bodies may themselves be implicated in the practices under scrutiny, such independence matters for trustworthy custody, rather than being an optional refinement. Finally, the large-scale destruction of eugenic-era records once their mandated retention periods had expired [2, 6] suggests that records-retention rules and archival policy should treat documentation of past injustice as a distinct category warranting active preservation and governed access rather than routine disposal [4, 21] — an implication relevant to regulatory and administrative decision-making. Together these points identify not a blueprint but the conditions — purpose-sensitivity, relational calibration, accountable and independent stewardship, and sustained public trust-building — under which the governance of such records is likely to be regarded as legitimate.

### Limitations and future directions

This study has several limitations. First, the survey addressed historical healthcare records more broadly rather than records related exclusively to the former Eugenic Protection Law; aggregate responses should not be read as views about specific record categories. Second, as reported in the Results, prior knowledge of the term “archives” was limited and varied by educational attainment; although an explanation was provided before the questionnaire, this variation in familiarity may have affected responses regarding custodial institutions. Third, the explanatory text presented before the questionnaire may have framed how respondents interpreted the concept of preservation institutions. The general-attitude items presented at the start of the survey may likewise have introduced context or priming effects. Fourth, the online survey design cannot fully capture respondents’ moral reasoning and concrete concerns.

Fifth, the participation rate (10.4%) was modest, and online consumer panels systematically under-represent individuals without regular digital access and those in long-term institutional care; the present findings therefore cannot represent the views of those most directly affected by the histories under examination. Because the achieved sample was somewhat more educated than the general population, the principal estimates were recomputed with the data reweighted to a range of national educational distributions; these estimates changed by no more than approximately 1% point, indicating that the descriptive findings are not driven by the sample’s educational composition (Additional file 2). Sixth, the study was designed primarily as a descriptive survey. The primary comparisons concerned variation by proximity and purpose, while demographic comparisons were exploratory. Because the demographic comparisons were exploratory and most did not remain significant after FDR correction — the principal exceptions being the educational gradient in acceptance and women’s greater concern about recalling painful experiences — subgroup findings should be interpreted as descriptive patterns rather than as evidence of independent predictors. In addition, the three proximity conditions were not a single graded scale. The self-or-living-family condition combined the respondent with living relatives in one item and cannot be disaggregated, and it differed from the ancestors condition in vital status and temporal distance as well as in personal closeness. The somewhat higher acceptance of research use for ancestral than for self-or-living-family records is therefore open to more than one interpretation, which the present data cannot adjudicate.

Finally, the study examined only general public attitudes and cannot represent the distinctive moral perspectives of survivors, affected families, and disability communities.

Several priorities follow from these findings and their limits. Most importantly, such general-population evidence needs to be complemented by deliberative and participatory work with these communities, whose participation is essential to legitimate governance [17–19, 31, 32, 35]. Comparative international studies would show how far the patterns observed here extend across the differing legal, archival, and cultural settings in which records of medical injustice are held, building on the German, Canadian, and U.S. experiences discussed above [30–34]. Qualitative and mixed-methods research — interviews, focus groups, and deliberative fora — could recover the public reasoning underlying the conditional, purpose- and proximity-sensitive attitudes documented here, which a questionnaire can register but not explain. Pursued together, these directions would extend the present work toward the participatory, cross-national, and interpretive evidence on which trustworthy governance of historical records of medical injustice ultimately depends.

## Conclusions

The case of historical healthcare records in Japan sharpens a familiar question in bioethics: where prospective consent is unavailable and those most directly recorded may no longer be reachable, the issue is not only how to use such records responsibly but whether their non-preservation is itself a form of injustice. The large-scale loss of records connected to the former Eugenic Protection Law suggests that it can be — obstructing verification, accountability, and redress — yet preservation and use carry their own risks of privacy infringement, renewed psychological harm, and the intergenerational reproduction of stigma. Such records can therefore be governed neither by preservation imperatives alone nor by consent-based research ethics; their legitimate governance depends on justifying use by purpose, protecting privacy in its relational as well as individual dimensions, and entrusting records to accountable stewards.

Public attitudes, as mapped here, do not settle these normative questions; they indicate the social conditions under which governance is likely to be regarded as legitimate — turning not only on whether records are preserved but on why they are used, how personally or familially proximate they are perceived to be, and who is entrusted with their custody. The task is thus not only to save records but to create justifiable conditions for their future use. The Japanese case examined here is best read not as a national exception but as an informative instance of a challenge shared internationally, one with clear counterparts in other societies confronting medical injustice in their past. This case illustrates why the governance of sensitive health information must extend beyond prospective consent — to the ethical afterlives of medical records created in contexts of injustice.

## Supplementary Information

Additional file 1: Survey questionnaire and explanatory materials. File format: DOCX. English translations of the full questionnaire as administered (including a small number of general-attitude and background items not analyzed in this study), together with summaries of the explanatory and framing materials shown to respondents, are provided.

Additional file 2: Cross-tabulations and association statistics. File format: XLSX. Aggregate analytic tables supporting the descriptive and exploratory analyses are provided, including cross-tabulations, chi-square statistics, Cramér's V, robustness and sensitivity analyses, and a Q-number mapping linking raw-data variables to questionnaire items.

## Data Availability

The anonymized survey dataset supporting the findings of this study is not publicly available because the data were generated through a third-party survey panel under contractual conditions that preclude unrestricted public sharing, and because respondents were not asked to consent to unrestricted public release of their data. Aggregate analytic tables, including cross-tabulations and association statistics, are provided in Additional file 2. Further de-identified aggregate data may be available from the corresponding author upon reasonable request and with the permission of INTAGE Inc., subject to ethical and contractual restrictions.

## Abbreviations

CARE: Collective Benefit, Authority to Control, Responsibility, and Ethics
CI: Confidence interval
FDR: False discovery rate
JST-RISTEX: Research Institute of Science and Technology for Society, Japan Science and Technology Agency
JSPS: Japan Society for the Promotion of Science
