# Supplementary material for: Governing healthcare records of past medical injustice beyond consent-based research ethics: a cross-sectional survey of public attitudes toward purpose, proximity, and stewardship in Japan
Source: BMC Med Ethics. 2026 Aug 12;27:231. doi: 10.1186/s12910-026-01571-8 (PMC13746683; doi:10.1186/s12910-026-01571-8)
Supplement: Supplementary file 1 [file 12910_2026_1571_MOESM1_ESM.docx]

**Additional file 1. Survey questionnaire and explanatory materials**

*Governing healthcare records of past medical injustice beyond consent-based research ethics: a cross-sectional survey of public attitudes toward purpose, proximity, and stewardship in Japan*

Survey conducted: 6–8 November 2024 / Valid responses: n = 1,143

**Note.** This questionnaire was administered in Japanese. The items below are English translations of the questions as administered, numbered consecutively for clarity. For readability, the three acceptance questions and the three purpose questions are grouped into Sections 4 and 5 respectively; as administered, each purpose question immediately followed the acceptance question for the same condition. The three proximity conditions appear in the order in which they were presented. The full instrument is reproduced, including a small number of general-attitude and background items (Section 1, Q1–Q3) that were collected but are not analyzed in this study. Respondent sex, age, and prefecture of residence were panel attributes provided by the survey company (INTAGE Inc.) and used for quota sampling; they were not asked as questionnaire items. Items whose options are marked ☐ are multiple-response items; items with lettered options (a, b, c, …) are single-response items. In Q3, the sub-items marked – were presented as a matrix, with the same response scale applied to each. In this additional file, “historical healthcare records” refers to records created in healthcare contexts, including those held by medical institutions, welfare facilities, and related public bodies. The explanatory and framing materials shown to respondents — the written description of archives, the background information on the preservation of historical healthcare records in Japan and abroad, and the accompanying illustrative images — are summarized at the start of the relevant sections below; their full original Japanese wording is available from the corresponding author on request.

**Section 1. General attitudes and background**

*The survey opened with items on respondents' prior use of record-holding facilities, their interest in news and topics concerning healthcare and welfare, and their general views on selected contemporary technologies. The following three items were collected first and are not analyzed in the present study; they are reproduced here for completeness and to indicate the order in which items were presented.*

**Q1.** Have you ever looked something up at a facility that preserves historical materials, such as a library, archive, or document repository?

**a** Yes, I have.

**b** No, I have not.

**Q2.** How interested are you in news and topics concerning healthcare and welfare?

**a** Interested.

**b** Somewhat interested.

**c** Not very interested.

**d** Not interested.

**Q3.** How do you generally feel about each of the following? Please select one option for each item. (Response scale: Acceptable / Rather acceptable / Rather unacceptable / Unacceptable.)

– COVID-19 vaccination

– Genetically modified (GM) crops

– Online administrative procedures using the My Number card

– Artificial intelligence (AI)-based facial-recognition security systems

**Section 2. Awareness of archives**

Respondents were first provided with a written explanation of what archives are, accompanied by photographs of an archival facility, before being asked the following question.

**Q4.** Before taking this survey, were you familiar with the term “archives” (archives/archival institutions)?

**a** I was familiar with the term and understood its meaning.

**b** I had heard the term but did not understand its meaning accurately.

**c** I had no prior knowledge of the term.

**Section 3. Attitudes toward preservation of healthcare records**

Respondents were provided with written background information on the preservation of historical healthcare records in Japan and other countries before answering the following questions.

**Q5.** Do you think historical healthcare records should be preserved over the long term?

**a** Should preserve.

**b** Rather should preserve.

**c** Rather should not preserve.

**d** Should not preserve.

**Q6.** [Displayed to respondents who selected “c” or “d” in Q5] What are your reasons for this view? Please select all that apply.

☐ I have concerns about privacy protection.

☐ Preservation incurs costs.

☐ I do not think such records are worth preserving.

☐ I am uncertain about what purposes the records might be used for in the future.

☐ Other

**Q7.** If historical healthcare records are to be preserved, how long do you think they should be kept? Please select the option closest to your view.

**a** Discard immediately (no preservation).

**b** Less than 10 years.

**c** 10 years.

**d** 50 years.

**e** 100 years.

**f** 150 years.

**g** Preserve permanently, without a fixed time limit.

**Q8.** If historical healthcare records are to be preserved, what type of institution do you think should be responsible for their custody? Please select the option that best reflects your view.

**a** The originating medical institution or welfare facility.

**b** The national archives.

**c** Local government archives or repositories.

**d** Universities or research institutions.

**e** Non-profit organizations.

**f** Private-sector organizations.

**g** Other

**Section 4. Acceptance of research use under different conditions**

The following three questions asked about research use of records under different conditions of personal proximity. Each was accompanied by illustrative examples of the type of records referred to.

**Q9.** We would like to ask about records held by medical institutions or welfare facilities that do not contain any personal information (e.g., statistics on hospital bed numbers, records of facility user counts, administrative records). What is your view on the use of such records for research purposes?

**a** Acceptable.

**b** Rather acceptable.

**c** Rather not acceptable.

**d** Not acceptable.

**Q10.** We would like to ask about records held by medical institutions or welfare facilities that contain information about yourself or your living family members (e.g., medical records created by a physician, case records from a welfare facility). What is your view on the use of such records for research purposes?

**a** Acceptable.

**b** Rather acceptable.

**c** Rather not acceptable.

**d** Not acceptable.

**Q11.** We would like to ask about records held by medical institutions or welfare facilities that contain information about your ancestors (e.g., a great-grandparent's medical record, healthcare records from 100 years ago). Here, “ancestors” refers to blood relatives from several or more generations ago who have already passed away. What is your view on the use of such records for research purposes?

**a** Acceptable.

**b** Rather acceptable.

**c** Rather not acceptable.

**d** Not acceptable.

**Section 5. Acceptable purposes of research use**

The following three questions asked about acceptable purposes of use under the same three conditions of personal proximity as above.

**Q12.** Regarding records that contain no personal information: if such records are to be used for research, what purposes would you consider acceptable? Please select all that apply.

☐ Medical research.

☐ Social welfare research.

☐ Humanities and social science research (e.g., history, sociology).

☐ Citizen-led inquiry.

☐ Journalistic investigations.

☐ Educational use (e.g., student research, classroom learning).

☐ None of the above.

**Q13.** Regarding records that contain information about yourself or your living family members: if such records are to be used for research, what purposes would you consider acceptable? Please select all that apply.

☐ Medical research.

☐ Social welfare research.

☐ Humanities and social science research (e.g., history, sociology).

☐ Citizen-led inquiry.

☐ Journalistic investigations.

☐ Educational use (e.g., student research, classroom learning).

☐ None of the above.

**Q14.** Regarding records that contain information about your ancestors: if such records are to be used for research, what purposes would you consider acceptable? Please select all that apply.

☐ Medical research.

☐ Social welfare research.

☐ Humanities and social science research (e.g., history, sociology).

☐ Citizen-led inquiry.

☐ Journalistic investigations.

☐ Educational use (e.g., student research, classroom learning).

☐ None of the above.

**Section 6. Perceived benefits and risks of research use**

The following two questions were asked in the context of research that draws on historical healthcare records to examine past ethical issues. Examples provided included: verification of non-consensual sterilizations carried out under the former Eugenic Protection Law; examination of the living conditions of patients institutionalized in Hansen's disease sanatoria; and review of initial responses to the COVID-19 pandemic.

**Q15.** What benefits do you think such research may have? Please select all that apply.

☐ Research based on such records can help society as a whole share lessons from the past.

☐ It can reveal historical realities that medical or welfare institutions might not wish to disclose on their own.

☐ It can contribute to the development of safer and fairer contemporary policies for patients and families.

☐ It can deepen understanding of the discrimination and prejudice experienced by patients and their families at the time.

☐ It can enable patients and their descendants to learn more accurately about the care and living conditions experienced in the past.

☐ It can increase opportunities for citizens and patients to learn about their own experiences.

☐ It can enable victims of medical human rights violations or pharmaceutical harms to draw on a wider range of materials in national compensation claims or civil litigation.

☐ None of the above.

**Q16.** What risks or drawbacks do you think such research may have? Please select all that apply.

☐ Past medical and welfare events are not personally relevant to me, so I have no interest in them.

☐ The costs of maintaining archival facilities are not justified by the research outcomes obtained.

☐ Clarifying past events may expose patients' families or descendants to discrimination or prejudice.

☐ There are concerns about privacy protection, and there is a risk of personal information being leaked.

☐ Some people may not wish to be reminded of painful past experiences, or may prefer the past to be forgotten.

☐ Governments, companies, or medical institutions may conceal or destroy information in order to avoid disadvantage or responsibility.

☐ Physicians or medical institutions may be held responsible for past events.

☐ None of the above.

**Section 7. Respondent characteristics**

*Sex, age, and prefecture of residence were provided by the survey company as panel attributes used for quota sampling (see Note above) and were not asked as survey items. The following demographic item was included in the questionnaire.*

**Q17.** What is the highest level of education you have completed?

**a** Middle school.

**b** High school.

**c** Vocational school.

**d** Junior/technical college.

**e** University.

**f** Graduate school.

**g** Other.

**h** Prefer not to answer.
